# Cognitive and Cerebral Aging Research in Autism: A Systematic Review on an Emerging Topic

**DOI:** 10.1002/aur.70031

**Published:** 2025-04-08

**Authors:** Marine Bessé, Shasha Morel‐Kohlmeyer, Emmanuelle Houy‐Durand, Philippe Prévost, Laurie Tuller, Badiâa Bouazzaoui, Laurence Taconnat, Julia Capdeville, Lucie Angel, Marie Gomot

**Affiliations:** ^1^ Centre de Recherches Sur la Cognition et l'Apprentissage (CeRCA), Team “Vieillissement et Psychopathologie de la Mémoire” UMR CNRS 7295, University of Tours University of Poitiers Tours France; ^2^ Université de Tours, INSERM, Imaging Brain & Neuropsychiatry iBraiN U1253 Tours France; ^3^ Centre Universitaire de Pédopsychiatrie, CHRU de Tours Tours France

**Keywords:** aging, autism, cerebral, cognitive, systematic review

## Abstract

Aging in autism is an emerging and under‐explored area of research. This systematic review provides a comprehensive overview of studies on cognitive and both structural and functional cerebral aging in autism. A systematic search of PubMed and APA PsycInfo was conducted up to and including January 2024. Two researchers independently screened and identified relevant English studies on cognitive (i.e., processing speed, executive function, working memory, episodic memory) and/or cerebral (i.e., structural and functional aspects) aging in autism. Study quality was assessed using the QualSyst quantitative scale to minimize bias. Thirty‐six studies met the inclusion criteria, with nine focusing on cerebral mechanisms, 19 on cognitive function, and eight addressing both. We examined cerebral and cognitive aging profiles in autism within the context of three hypotheses: accelerated aging, parallel aging, and the safeguard hypothesis. The synthesis does not reveal a consistent pattern with respect to any of the three hypotheses, as results varied across methodology types (cross‐sectional vs. longitudinal) and studies, even with similar measures of cerebral or cognitive function. This systematic review highlights the ongoing lack of consensus in this area, which may be attributed to various internal or external factors (e.g., participants age, co‐occurring conditions, lifestyle, cognitive reserve). Despite divergent findings, this review suggests that cross‐sectional studies on cerebral and cognitive autistic aging predominantly align with the parallel or safeguard hypothesis. In contrast, the few longitudinal studies, which are the only ones capable of directly informing the aging process, are more consistent with the parallel or accelerated hypothesis. Further research is crucial to understand how cerebral and cognitive aging impact autistic symptomatology, enabling tailored support.

## Summary

- This review explores current research on cognitive and cerebral aging in autism, mostly characterized by mixed findings. Studies comparing groups of people of different ages suggest that the effects of age on cognitive and cerebral functioning may be similar, or even less pronounced in autistic compared to non‐autistic adults.
- In contrast, studies following individuals over time (longitudinal studies) indicate that aging might have a similar or more significant impact on autistic individuals.
- Further research is essential to better understand how these aging processes affect autism symptoms and to ensure that autistic adults receive the necessary support as they age.

## 1 Introduction

Over the past 30 years, there has been a notable increase in the global aging population, with an estimated rise from 13.7% in 2021 to 22% of people aged over 60 years by 2050 (World Health Organization 2022). Consequently, the number of autistic adults entering old age is also expected to rise significantly (Mukaetova‐Ladinska et al. 2012). With autism prevalence estimated at 1% (Zeidan et al. 2022), this could result in over 21 million autistic individuals aged 60 and older worldwide within the next 30 years. Epidemiological studies suggest a slight decrease in autism prevalence with advancing age: 16–44 years, 1.1%; 45–74 years, 0.9%; +75 years, 0.8% (Brugha et al. 2009, 2011). This decline has been associated with a reduced life expectancy in autistic individuals, particularly those with co‐occurring neurological or genetic conditions or intellectual developmental disorders (IDD) (Perkins and Berkman 2012). Nevertheless, this prevalence might be underestimated (van Niekerk et al. 2011) due to the inclusion of autism in the DSM‐III classification manual of mental disorders only in 1980 and evolving diagnostic practices that may have led to missed diagnoses in older generations (American Psychiatric Association 1980). Diagnosing autism in middle‐aged and older adults remains challenging due to inadequate diagnostic tools and limited historical developmental information (e.g., ADI‐R) (van Niekerk et al. 2011). Ultimately, the growing number of autism diagnoses suggests an even larger future population of autistic adults (Piven and Rabins 2011).

Autism's complexity and severity evolve throughout the lifespan, with some studies indicating that middle‐aged individuals exhibit fewer autistic symptoms than in childhood (Esbensen, Seltzer, et al. 2009; Hong et al. 2023; Howlin et al. 2013), with reductions in both restricted and repetitive behaviors (Esbensen, Seltzer, et al. 2009; Howlin et al. 2013), and greater social reciprocity and verbal communication (Hong et al. 2023). However, these studies included few adults over 65 years, leaving knowledge about the aging consequences limited. Aging in autism is shaped by both general aging processes and autism‐specific characteristics. As individuals age, changes in sensory, cognitive, and brain functions (e.g., Park 2002; Peelle 2019; Raz 2000) may influence autistic symptomatology, which may evolve differently across individuals. The presence of additional co‐occurring conditions such as IDD or neurological and psychiatric disorders may also add to this variability. While recent research suggests that the prevalence of co‐occurring conditions (e.g., anxiety, mood disorders, aggressive behaviors) may be less pronounced in older autistic individuals (Fortuna et al. 2016; Lever and Geurts 2016b; Uljarević et al. 2020; Wise et al. 2017; Yarar et al. 2022), our understanding of how these conditions interact with aging remains limited. Additionally, autistic adults face a higher risk of neurodegenerative diseases, such as Parkinson's (27% in autistic adults vs. 2.3% in the general population; Starkstein et al. 2015) and Alzheimer's (4.04% in autistic adults without IDD vs. 0.97% in the general population; Vivanti et al. 2021). Similarly, Vivanti et al. (2025) reported a high prevalence of dementia diagnoses (35.12%) among older autistic adults (≥ 65 years) enrolled in US Medicaid and Medicare. This increased risk of neurodegenerative diseases emphasizes the need for a deeper exploration of how cerebral and cognitive mechanisms develop and change over time in autism.

Bowler (2007) proposed the “aging analogy” to highlight the striking similarities between the cognitive functioning of autistic adults and the typical aging process. This analogy also extends to cerebral functioning (Bowler 2017) and raises questions about the cumulative effects of autism and aging on cognitive and brain functioning. Three hypotheses characterize the cognitive aging process in autism (Geurts and Vissers 2012), which are well‐described in a recent meta‐analysis (Wang et al. 2024) and extend to cerebral aging as well. The parallel aging hypothesis suggests that autistic individuals age similarly to non‐autistic adults, with either comparable or consistently lower cognitive performance, but the overall aging trajectory remains parallel. The accelerated aging hypothesis posits that cognitive difficulties in autistic adults may intensify with age, leading to earlier or faster cognitive aging. The safeguard aging hypothesis proposes that autistic adults, facing cognitive challenges from a young age, may age more slowly due to compensatory strategies developed over their lives.

Despite increasing focus on aging in autistic individuals, research on this topic remains minimal, representing just 0.4% of overall autism research (Mason et al. 2022). Indeed, although there is extensive research on cognitive and brain development during childhood in autism, our knowledge of how cognitive and cerebral features evolve in later adulthood remains limited. This underscores the urgent need to enhance our understanding of the clinical, cognitive, and cerebral aspects of aging in this population (Happé and Charlton 2011; Wise 2020; Wright et al. 2013). While previous studies, including a systematic review that explored the characteristics of old‐aged autistic adults, included only some cognitive aspects (Tse et al. 2022) and a meta‐analysis focusing on cognitive and only on brain morphological deviations in middle‐to‐old‐aged autistic adults (Wang et al. 2024), have provided valuable insights, the present review is the first to comprehensively analyze cognitive and both structural and functional cerebral aging processes in this population. This review includes both cross‐sectional and longitudinal designs, which are presented separately to highlight key differences. This separation stems from the limitations of cross‐sectional studies, particularly their inability to accurately capture aging trajectories compared to longitudinal designs, as well as the potential influence of cohort differences within the autistic population on the findings. We examine non‐social cognitive functions such as processing speed, executive function, working memory, and episodic memory, which are particularly affected by aging. Additionally, we explore structural changes in brain volume, cortical thickness, and white matter integrity, along with functional changes in brain activation, connectivity, and electrophysiological indicators. This review, by integrating previously unaddressed functional aspects, aims to provide a complete understanding of aging effects in autism, identify research gaps, and offer novel perspectives for future research.

## 2 Method

### 2.1 Search Strategy

This systematic review, following PRISMA guidelines (Page et al. 2021), included a comprehensive search of PubMed and APA PsycInfo up to and including January 2024 and manually identified all studies on cognitive and/or cerebral aging in autistic individuals. PubMed and PsycInfo search details are listed in Table 1. We selected PubMed and APA PsycInfo for their comprehensive coverage of neuroscientific and cognitive psychological literature relevant to this review, while excluding broader databases like Web of Science or SCOPUS due to significant overlap. Titles, keywords, or abstracts had to include at least one term from the topics: (Autism AND Aging AND Cognitive) OR (Autism AND Aging AND Cerebral). Additional searches were performed by reviewing references cited in the identified studies.

**TABLE 1:** PubMed and PsycInfo search details.

Pubmed
((Autis*[Title/Abstract] OR ASD[Title/Abstract] OR Asperger[Title/Abstract]) **AND** (Aging[Title/Abstract] OR Ageing[Title/Abstract] OR Older Adult*[Title/Abstract] OR Elder*[Title/Abstract] OR Senior*[Title/Abstract] OR Middle Age*[Title/Abstract]) **AND** (Cogni*[Title/Abstract] OR Memor*[Title/Abstract] OR Executi*[Title/Abstract] OR Speed*[Title/Abstract])) **OR** ((Autis*[Title/Abstract] OR ASD[Title/Abstract] OR Asperger[Title/Abstract]) **AND** (Aging[Title/Abstract] OR Ageing[Title/Abstract] OR Older Adult*[Title/Abstract] OR Elder*[Title/Abstract] OR Senior*[Title/Abstract] OR Middle Age*[Title/Abstract]) **AND** (Cerebral*[Title/Abstract] OR Brain*[Title/Abstract] OR Volume*[Title/Abstract] OR Structural*[Title/Abstract] OR Thickness*[Title/Abstract] OR Gray Matter*[Title/Abstract] OR White Matter*[Title/Abstract] OR Functional*[Title/Abstract] OR EEG[Title/Abstract] OR Electrophysio*[Title/Abstract] OR Network*[Title/Abstract]))

PsycInfo
((Autis* OR ASD OR Asperger) **AND** (Aging OR Ageing OR Older Adult* OR Elder* OR Senior* OR Middle Age*) **AND** (Cogni* OR Memor* OR Executi* OR Speed*)) **OR** ((Autis* OR ASD OR Asperger) **AND** (Aging OR Ageing OR Older Adult* OR Elder* OR Senior* OR Middle Age*) **AND** (Cerebral*OR Brain* OR Volume* OR Structural* OR Thickness* OR Gray Matter*OR White Matter* OR Functional* OR EEG OR Electrophysio* OR Network*))

### 2.2 Eligibility Criteria

Inclusion criteria were: (1) studies that included middle‐aged (over 40 years) or older autistic participants (over 60 years), (2) studies with participants clinically diagnosed with autism by a medical or clinical professional based on established diagnostic criteria (e.g., DSM‐IV/DSM‐V; ICD‐10), (3) studies focusing on cognitive outcomes (i.e., processing speed, executive functions, working memory, and episodic memory) or cerebral outcomes (i.e., including structural and functional cerebral aspects), (4) studies written in English.

Exclusion criteria included: (1) non‐primary sources (e.g., meta‐analysis, systematic review, book chapters, case report), (2) animal research studies, (3) studies not assessing the target population (e.g., young autistic adults only, the broad autism phenotype, other psychiatric disorders, other genetic disorders), (4) studies unrelated to outcomes (e.g., social cognition (e.g., theory of mind, language), or molecular mechanisms (e.g., gene expression, neuropeptides, neurotransmitters)), and (5) post‐mortem studies.

### 2.3 Article Selection

**FIGURE 1:**
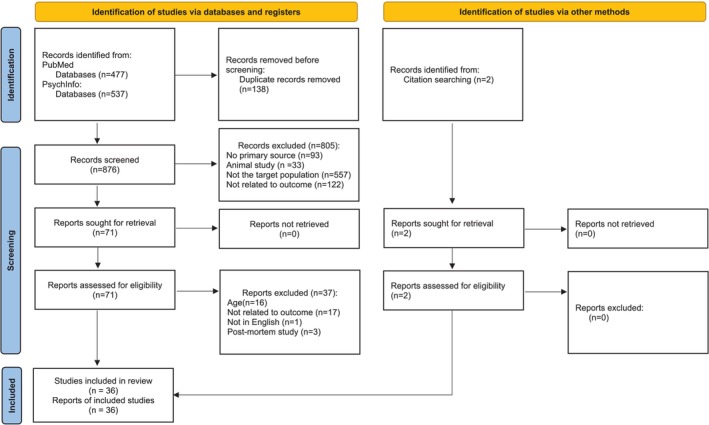
PRISMA flow diagram.

PubMed and PsycInfo searches yielded 477 and 537 articles, respectively, with an additional two articles identified through reference inspection. After removing duplicates, the titles and abstracts of the 876 remaining articles were reviewed for inclusion criteria. To guarantee consistency, two authors (M.B. and J.C.) independently screened titles and abstracts and selected studies for inclusion. Disagreements were resolved through consensus meetings. This process led to the exclusion of 805 articles, leaving 73 for further review. The first author (M.B.) then screened the full texts of these 73 articles for compliance with inclusion criteria, focusing on study design, participant characteristics, and methods used to assess cognitive or cerebral functioning. Each study was reviewed to ensure it met the eligibility criteria for the specific syntheses, including only those with relevant data. Most studies were excluded for not meeting age criteria (*n* = 16) or focusing on irrelevant measures (e.g., social cognition, molecular mechanisms) (*n* = 17). Additionally, one study was excluded because it was not written in English, and three post‐mortem studies were removed. Ultimately, 36 studies were retained for review. The study selection procedure is illustrated in Figure 1. The studies are categorized into two main focus groups for a structured synthesis of evidence:

1. Cerebral functioning: This group includes studies examining structural and functional brain aspects related to aging in autism, including comparisons between autistic and non‐autistic individuals.

2. Cognitive functioning: This group includes studies assessing cognitive outcomes such as processing speed, executive functions, working memory, and episodic memory, to identify patterns and differences in cognitive functioning during aging between autistic and non‐autistic participants.

### 2.4 Data Extraction

The first author (M.B.) manually extracted data from each study, focusing on key elements, such as: (1) Study design (cross‐sectional or longitudinal), (2) Clinical assessment of autism diagnosis, (3) Number of participants, (4) Sex ratio, (5) Mean age and age range of participants, (6) IQ level, (7) Cerebral assessment, (8) Main cerebral findings, (9) Cognitive assessment, and (10) Main cognitive findings. We aimed to include all relevant results for each predefined outcome domain but included only the main findings to ensure clarity and focus. Main findings were defined as outcomes directly relevant to the predefined domains of cognitive and cerebral functioning, including both significant and non‐significant results, as well as conclusions that align with the focus of this review. This approach ensured a comprehensive assessment while presenting results in a clear and concise manner.

### 2.5 Synthesis Method

Data from each study were compiled into detailed tables outlining key characteristics of cerebral (Table 2) and cognitive functioning (Table 3), including study design, participant demographics, cerebral and/or cognitive measures, and main findings. Studies that evaluated both cerebral and cognitive measures are summarized in Table 2. This organization facilitated a thorough evaluation and coherent analysis of the studies. We conducted a qualitative synthesis by summarizing and comparing findings across studies to draw meaningful insights and identify patterns.

**TABLE 2:** Characteristics of structural and functional brain and cognitive aging studies in autism.

Author (year)	Clinical assessment	*N* (male)	Age (Y) Mean (SD) [Range]	IQ (SD)	Measure(s)	Main findings	QualSyst score
Cross‐sectional studies							
Bathelt et al. (2020)	ASD (Clinical diagnosis of ASD; ADOS ≥ 7 and/or AQ ≥ 26	ASD: 44 (31) NT: 45 (30)	ASD: 50.6 (12.21) [30–74] NT: 48.9 (11.34) [30–74]	ASD: 116.43 (16.89) NT: 111.13 (15.41)	Functional connectivity fMRI: Resting state sequence; Social processing paradigm task; Flanker‐type response inhibition paradigm task	Diagnosis: ASD>NT for default mode network activity; ASD = NT for visual and frontoparietal control network Age: 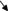 with age for the connection strengh of the default mode network Age × Diagnosis: Less negative connection strengh of the visual‐frontal parietal control network with age only in the NT group	0.86 (19/22)
Baxter et al. (2019)	ASD (DSM‐V; ADOS)	YA: ASD: 18 men NT: 14 men MAA: ASD: 24 men NT: 20 men	YA: (18–25) ASD: 21 (3) NT: 21 (3) MAA: (40–60) ASD: 53 (8) NT: 50 (7)	YA: ASD: 105.33 (13.5) NT: 107.21 (12.28) MAA: ASD: 106.00 (17.93) NT: 112.50 (12.79)	Functional MRI: During a fluency task Processing speed: Word reading trial of the Stroop task Trail Making Task, part A	Diagnosis: ASD<NT for default mode network, the posterior cingulate^b^ and the left angular gyrus activity^b^ during the fluency task and for processing speed. ASD=NT for the performances of the fluency task Age: YA>MAA for the left thalamus activity; YA<MAA for the supplementary speech area and superior temporal activity^b^. No age effect observed for processing speed Age × Diagnosis: Distinct networks activation between the four groups during the fluency task. No interaction with age observed for processing speed	0.95 (21/22)
Braden et al. (2017)	ASD (DSM IV or DSM V; adOS ≥ 7)	ASD: 16 (16) NT: 17 (17)	ASD: 50.10 (1.7) [40–62] NT: 50.00 (1.8) [40–64]	ASD: 108.9 (3.4) NT: 110.2 (2.7)	Functional connectivity MRI: during a verbal working memory task (0‐, 1‐, and 2‐Back task) Structural connectivity MRI: FA and MD Structural MRI: Hippocampal volumes Episodic memory: Verbal memory (RAVLT) Executive functions: *Flexibility*: Wisconsin Card Sorting Task	Diagnosis: ASD = NT for working memory network, default mode network and salient network, for FA and MD, and for verbal memory and working memory; ASD<NT for cortico‐striato‐thalamo‐cortical neural network, for hippocampal volumes and for cognitive flexibility Age: no impact of age for any measures (measured only in ASD)^a^	
Braden and Riecken (2019)	ASD (DSM‐IV‐TR; ADOS and/or ADI)	ASD: 157 (144) NT: 172 (154)	ASD: 30.9 (12.4) [18–64] NT: 28.8 (10.5) [18–64]	ASD: 109.0 (15.3) NT: 113.9 (10.9)	Structural MRI: CT	Diagnosis: ASD = NT for CT Age: 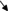 with age for CT in frontal, temporal, and parietal lobe, 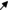 with age for CT in medial temporal regions Age × Diagnosis: 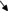 with age for CT in left frontal lobe, temporal lobe, parietal and lateral occipital lobe more pronounced in the ASD group	0.82 (18/22)
Dickinson et al. (2022)	ASD (DSM‐IV; DSM‐V; ICD‐10; SRS; ADOS)	ASD: 93 (67) NT: 87 (53)	ASD: 30.39 (13.64) [18–67] NT: 32.52 (12.30) [18–69]	ASD (NVIQ): 52.64 (10.21) NT (NVIQ): 57.16 (9.20)	Functional EEG: Peak Alpha Frequency (PAF) indicator	Diagnosis: ASD>NT for NVIQ level. ASD=NT for the relation between PAF and NVIQ level Age: 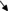 with age for PAF Age × Diagnosis: 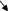 with age for PAF more pronounced in the ASD group	0.86 (19/22)
Hau et al. (2021)	ASD (DSM‐V; ADOS‐2)	ASD: 26 (21) NT: 26 (23)	ASD: 51.1 (6.0) [41–64] NT: 51.6 (6.1) [40–60]	ASD: 106 (22) NT: 116 (12)	Structural connectivity MRI: FA, MD, RD, and AD	Diagnosis: ASD=NT for the white matter integrity of corticospinal tract Age: no age effect observed Age × Diagnosis: no interaction observed	0.91 (20/22)
Hau et al. (2022)	ASD (DSM‐V; ADOS‐2)	ASD: 25 (20) NT: 24 (21)	ASD: 51.7 (6.9) [41–67] NT: 51.6 (7.0) [41–70]	ASD: 106 (23) NT: 115 (12)	Structural MRI: CV and CT Structural connectivity MRI: FA and MD	Diagnosis: ASD>NT for CV in the right precentral hand knob^b^ and for MD in the post and precentral hand knob^b^; ASD<NT for the functional connectivity in the right post and precentral hand knob^b^; ASD=NT for the CT in the precentral hand knob and FA for the post and precentral hand knob Age: not reported Age × Diagnosis: 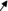 with age in the ASD group and 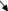 with age in the NT group for CV of the right precentral hand knob^b^; 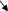 with age in the ASD group and 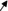 with age in the NT group for the functional connectivity of the left post and precentral hand knob^b^	0.91 (20/22)
Kohli et al. (2019)	ASD (DSM‐V; ADOS‐2)	ASD: 20 (16) NT: 21 (20)	ASD: 50.2 (5.9) [41–60] NT: 50.8 (6.9) [40–60]	ASD: 101.55 (27.76) NT: 120.90 (11.85)	Structural MRI: CT Executive functions: *Flexibility*: D‐KEFS Trails task *Inhibition*: D‐KEFS Color Word Interference task	Diagnosis: ASD=NT for CT; ASD<NT for flexibility and inhibition Age: 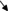 with age for CT across the cortex Age × Diagnosis: no interaction observed	0.77 (17/22)
Koolschijn and Geurts (2016)	ASD (clinical diagnosis; adOS ≥ 7 or AQ ≥ 26)	ASD: 51 (35) NT: 49 (32)	ASD: 51.46 (12.61) [30–74] NT: 50.14 (11.94) [30–74]	ASD: 116.31 (16.21) NT: 111.59 (15.78)	Structural MRI: GM volume and CT	Diagnosis: ASD=NT for GM volume and CT Age: 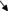 with age for GM volume and CT. Age × Diagnosis: no interaction observed	0.95 (21/22)
Koolschijn et al. (2017)	ASD (clinical diagnosis; ADOS ≥ 7 or AQ ≥ 26)	ASD: 48 (33) NT: 48 (31)	ASD: 51.32 (12.29) [30–74] NT: 50.47 (11.83) [30–74]	ASD: 116.60 (16.04) NT: 110.98 (15.34)	Structural connectivity MRI: FA, MD, RD, and AD Executive functions: *Inhibition*: Flanker task	Diagnosis: ASD>NT for MD and RD. Negative association between MD/RD and the performance in the cognitive task only in ASD. ASD=NT for inhibition. Age × Diagnosis: 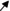 with age for MD and RD in the ASD group	0.86 (19/22)
Linke et al. (2020)	ASD (DSM‐V; ADOS‐2)	ASD: 17 (14) NT: 19 (18)	ASD: 49.7 (6.6) [40–60] NT: 50.4 (6.3) [40–60]	ASD: 102 (24) NT: 120 (11)	Functional connectivity MRI: During resting state	Diagnosis: ASD<NT for the connectivity within the sensorimotor system Age: no age effect observed for the connectivity of the sensorimotor system^a^	0.82 (18/22)
Raznahan et al. (2010)	ASD (ICD‐10; ADI‐R; ADOS‐G)	ASD: 76 (76) NT: 51 (51)	ASD: 31.7 (12.1) [10–60] NT: 28.6 (12.6) [11–59]	ASD: 105 (16.5) NT: 122 (16.8)	Structural MRI: CV and CT	Diagnosis: not reported. Age: 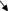 with age for CV and CT Age × Diagnosis: 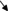 with age for CV and CT especially in frontal (only for CT) and temporal regions only in the NT group	0.82 (18/22)
Tung et al. (2021)	ASD (DSM‐V; ADOS‐2)	ASD: 22 (16) NT: 26 (4)	ASD: 49.45 (6.03) [40–60] NT: 51.0 (7.03) [40–64]	ASD: 104.32 (23.41) NT: 116.4 (11.01)	Structural MRI: CV Functional connectivity MRI: During resting state	Structural MRI. Diagnosis: ASD=NT for CV Functional connectivity MRI. Diagnosis: ASD<NT within the anxiety network^a^	0.91 (20/22)
van Rooij et al. (2018)	ASD (Clinical diagnosis; DSM‐IV)	ASD: 1571 (1347) NT: 1651 (1258)	ASD: 15.41 (8.64) [2–64] NT: 15.83 (8.41) [2–56]	ASD: 103 (20.02) NT: 111 (19.04)	Structural MRI: Subcortical volume, CT	Diagnosis: ASD>NT for CT, volume of lateral ventricles, intracranial volume; ASD<NT for the volume of the putamen, pallidum, amygdala and nucleus accumbens Age: 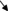 with age for all subcortical volume and CT Age × Diagnosis: ASD>NT for CT only around adolescence; No interaction observed for subcortical volume	0.82 (18/22)
Walsh et al. (2019)	ASD (DSM‐V; ADOS‐2; self‐reported psychiatric history interview)	YA: ASD: 24 men NT: 15 men MAA: ASD: 25 men NT: 21 men	YA: (18–25) ASD: 21.1 (2.3) NT: 20.9 (2.4) MAA: (40–70) ASD: 53.0 (8.8) NT: 49.7 (6.9)	YA: ASD: 104.60 (14.4) NT: 111.80 (13.1) MAA: ASD: 109.60 (15) NT: 111.00 (13.5)	Functional connectivity MRI: During resting state Executive functions: *Planning*: Tower of London	Diagnosis: ASD=NT within the executive network. Not reported for planning Age: no age effect observed within the executive network. Not reported for planning Age × Diagnosis: Hyppoconnectivity of the left dlPFC of the executive network more pronounced in the older ASD group. No differences between groups for planning	0.86 (19/22)
Longitudinal (and cross‐sectional) studies							
Pagni et al. (2022)	ASD (DSM‐V; ADOS‐2 ≥ 7)	CSS: ASD: 106 (74) NT: 89 (52) LS: ASD: 23 (19) NT: 22 (18)	CSS: ASD: 39.58 (16.02) [18.71] NT: 40.94 (16.72) [18–70] LS: ASD: 52.57 (8.46) [40–66] NT: 50.45 (7.05) [40–64]	CSS: ASD: 106.71 (14.48) NT: 108.28 (11.99) LS: ASD: 110.57 (14.17) NT: 111.18 (14.14)	Structural MRI: Hippocampal volumes Structural connectivity MRI: FA Episodic memory: Verbal memory (RAVLT)	CSS: Diagnosis: ASD<NT for FA and for delayed verbal memory; ASD=NT for hippocampal volume and for immediate verbal memory Age: 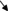 with age for hippocampal volumes, FA, and for immediate and delayed verbal memory Age × Diagnosis: 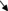 with age for immediate verbal memory only in the NT group. No other interaction observed LS: Diagnosis: ASD<NT for hippocampal volumes and FA; ASD=NT for delayed verbal memory. Not reported for immediate verbal memory Change over time: 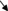 over time for FA only Change over Time × Diagnosis: 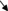 over time for hippocampal volume and immediate verbal memory more pronounced in ASD group; no change over time observed for FA and delayed verbal memory	0.91 (20/22)
Walsh et al. (2022)	ASD (DSM‐V; ADOS ≥ 7)	ASD: 25 (21) NT: 25 (21)	ASD: 52.60 (8.18) [40–66] NT: 50.00 (6.78) [40–64]	ASD: 111.00 (13.66) NT: 110.80 (13.53)	Structural MRI: Hippocampal volumes Structural connectivity MRI: FA and free‐water Episodic memory: Visual memory (WMS‐III)	Diagnosis: ASD<NT for hippocampal volumes, FA and for delayed visual memory; ASD=NT for free‐water and immediate visual memory Age: No effect of age for immediate verbal memory Change over time: 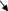 over time for delayed visual memory; no change over time for immediate verbal memory Change over Time × Diagnosis: 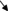 over time for delayed visual memory more pronounced in the ASD group; no interaction observed for immediate verbal memory Correlation: Baseline hippocampal free‐water negatively correlated with delayed visual memory change over time in the ASD group.	0.95 (21/22)

Abbreviations: AD: axial diffusivity; ADI(−R): autism diagnostic interview (‐revised); ADOS: autism diagnostic observation schedule; AQ: autism spectrum quotient; ASD: autism spectrum disorder group; CSS: cross sectional study; CT: cortical thickness; CV: cortical volume; DSM‐IV/DSM‐V: diagnostic and statistical manual of mental disorders fourth/fifth edition; EQ: empathy quotient; FA: fraction of anisotropy; GM: gray matter volume; ICD‐10: International Classification of Diseases 10th Revision; IQ: intelligent quotient; LS: longitudinal study; MAA: middle‐aged adults; MD: mean diffusivity; NT: neurotypical group; RD: radial diffusivity; SD: standard deviation; SQ: systemizing quotient; YA: young adults.

^a^ Interaction not performed.

^b^ Effect that did not survive FDR correction.

**TABLE 3:** Characteristics of cognitive aging studies in autism.

Author (year)	Clinical assessment	*N* (male)	Age (Y) Mean (SD) [Range]	IQ (SD)	Measure(s)	Main findings	QualSyst Score
Cross‐sectional studies							
Abbott et al. (2018)	ASD (ICD‐10; AQ; EQ; SQ)	ASD: 134 (97)	ASD: 31.14 (11.94) [18–75]	IQ > 70	Executive functions and processing speed: *Processing speed*: Digit symbol (WAIS III–IV) *Initiation/inhibition*: Hayling test *Flexibility*: Trails A and B (AITB) *Planning*: Zoo map, Key search (BADS) Working memory: Digit span (WAIS III–IV)	Age: 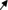 with age for processing speed and flexibility. No effect of age observed for initiation/inhibition, planning and working memory	0.91 (20/22)
Charlton et al. (2023)	ASD (clinical diagnosis; AQ28)	ASD: 350 (147) NT: 350 (109)	ASD: 51.66 (8.91) [40–83] NT: 52.75 (9.1) [40–79]	Not reported	Episodic memory: Prospective, Retrospective memory questionnaire (PRMQ)	Diagnosis: ASD>NT for self‐reporting memory difficulties Age: 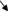 with age for self‐reporting memory difficulties only in the ASD group (separate correlational analysis between groups)^a^	0.82 (18/22)
Davids et al. (2016)	ASD: (clinical team; DSM‐IV or DSM‐V; ADOS)	ASD: 36 (30) NT: 36 (30)	ASD: 58.6 (7.8) [50–84] NT:59.4 (8.3) [50–79]	ASD: 106.3 (18.4) NT: 107.1 (15.6)	Executive functions: *Subjective executive functioning*: BRIEF‐A *Planning*: Tower of London and Zoo map (BADS) Processing speed: Processing speed index (WAIS‐IV)	Diagnosis: ASD>NT for self‐reporting executive function difficulties; ASD=NT for all the other measures Age: No age effect observed^a^	0.95 (21/22)
Davids et al. (2020)	ASD: (clinical diagnosis of autism)	ASD: 36 (30) NT: 36 (30)	ASD: 58.6 (7.8) [50–84] NT:59.4 (8.3) [50–79]	ASD: 106.3 (18.4) NT: 107.1 (15.6)	Episodic memory: Visual memory: Rey Osterrieth Complex Figure Test Processing speed: Processing speed index (WAIS‐IV) Working memory: Working memory index (WAIS‐IV)	Diagnosis: ASD=NT for all the measures^a^	0.91 (20/22)
Geurts and Vissers (2012)	ASD (clinical diagnosis of autism and/or SRS > 60)	ASD: 23 (18) NT: 23 (18)	ASD: 63.6 (7.5) [51–83] NT: 63.7 (8.1) [51–83]	ASD: 109.5 (10.3) NT: 109.8 (7.9)	Episodic memory: Visual memory (WMS‐III) Verbal memory (RAVLT) Working memory: Spatial span (WMS‐III) Executive functions: *Flexibility*: Modified Card Sorting Test and Trail Making Test *Planning*: Tower of London Processing speed: Digit Symbol‐copy (WAIS‐III)	Diagnosis: ASD<NT for working memory; ASD=NT for all the other measures. Age: 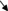 with age for delayed visual memory and immediate verbal memory. No effect of age observed for the other measures Age × Diagnosis: 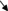 with age for immediate visual memory more pronounced in the ASD group. No interaction observed for the other measures	0.86 (19/22)
Geurts et al. (2020)	ASD (clinical interview; DSM‐IV; AQ)	ASD: 50 (not reported) NT: 51 (not reported)	ASD: 65.8 (5.6) [60–85] NT: 69.7 (5.6) [60–83]	ASD: 110.7 (12.2) NT: 110.7 (12.5)	Executive functions: *Subjective executive functioning*: BRIEF‐A *Planning*: D‐KEFS Tower Task and Zoo map (BADS) *Flexibility*: Wisconsin Card Sorting Task Processing speed: Processing speed index (WAIS‐IV) Working memory: Working memory index (WAIS‐IV)	Diagnosis: ASD>NT for self‐reporting executive function difficulties. ASD=NT for all the other measures Age: no impact of age for any measures Age × diagnosis: no interaction for any measures	0.91 (20/22)
Happé et al. (2016)	ASD (clinical diagnosis; AQ; EQ; SQ)	ASD: 95 (not reported) NT: 41 (not reported)	ASD: 30.02 (10.77) [18–74] NT: 37.20 (14.36) [18–67]	IQ > 70	Processing speed: *Executive control, processing speed*: Digit symbol (WAIS III‐IV)	Diagnosis: ASD=NT for processing speed Age: 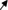 with age for processing speed only in the ASD group (separate correlational analysis between groups)^a^	0.77 (17/22)
Harker et al. (2023)	ASD (clinical diagnosis; DSM‐V; ADOS)	ASD: 35 (27) NT: 41 (27)	ASD: 53.06 (8.91) [40–71] NT: 53.90 (8.44) [40–70]	ASD: 108.97 (14.52) NT: 109.07 (12.09)	Episodic memory: Verbal memory (RAVLT)	Diagnosis: ASD=NT for verbal memory	0.91 (20/22)
Lever et al. (2015)	ASD (DSM‐IV; ADOS; AQ)	ASD: 111 (79) NT: 164 (93)	ASD: 47.5 (15.0) [20–79] NT: 46.0 (16.5) [19–77]	ASD: 115.2 (16.9) NT: 113.3 (16.7)	Executive functions: *Updating*: N‐back task in three level (0‐back, 1‐back and 2‐back)	Diagnosis: ASD<NT only for reaction time measure Age: 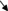 with age for updating capacity Age × Diagnosis: 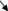 with age for updating capacity especially in the NT group	0.86 (19/22)
Lever and Geurts (2016a)	ASD (DSM‐IV; ADOS ≥ 7; AQ ≥ 26)	ASD: 118 (83) NT: 118 (83)	ASD: 47.6 (14.9) [20–79] NT: 47.7 (15.4) [20–77]	ASD: 114.8 (16.9) NT: 114.3 (15.3)	Episodic memory: Visual memory (WMS‐III) Verbal memory (RAVLT)	Diagnosis: ASD>NT for immediate visual memory, ASD=NT for verbal memory Age: 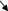 with age in visual and verbal memory Age × Diagnosis: 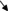 with age for immediate and recognition visual memory more pronounced in the NT group	0.95 (21/22)
Powell et al. (2017)	ASD (clinical interview; ADOS‐2 ≥ 6; SRS‐2 ≥ 65)	ASD: 29 (24) NT: 30 (23)	ASD: 49.0 (11.7) [30–67] NT: 48.7 (12.1) [30–65]	ASD: 113.2 (9.5) NT: 113.1 (10.2)	Processing Speed and Executive Functions (flexibility) Visual scanning, number sequencing and letter sequencing subtests of the Trail Making Test Episodic memory: Verbal memory (RAVLT)	Diagnosis: ASD<NT for verbal memory, processing speed, ASD=NT for flexibility Age: 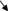 with age for verbal memory, processing speed and flexibility Age × Diagnosis: 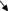 with age for flexibility only in the ASD group. No other interaction observed	0.91 (20/22)
Ring et al. (2016)	ASD (DSM‐IV; ADOS)	ASD: 18 (13) NT: 18 (14)	ASD: 42.78 (11.8) [20–62] NT: 43.48 (13.0) [23–61]	ASD: 108 (17.9) NT: 109 (17.2)	Episodic memory: Relational memory (Item vs. Location test vs. Order test vs. Associative test)	Diagnosis: ASD<NT for all measures Age: 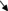 with age for the order task Age × Diagnosis: 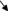 with age for the order task only in the NT group	0.77 (17/22)
Ring et al. (2020)	ASD (DSM‐IV; ADOS)	ASD: 53 (42) NT: 52 (38)	ASD: 43.66 (12.64) [25–65] NT: 42.40 (12.73) [21–67]	ASD: 113 (16.61) NT: 114 (14.97)	Working memory: Verbal span task, Visuospatial span task, Multimodal span task and Integration task Executive function: *Flexibility*: Color Trails Test	Diagnosis: ASD<NT for all measures Age: 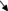 with age for the visual and multimodal span task Age × Diagnosis: 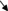 with age for the visual and multimodal span task only in the NT group EF and visual and multimodal span task difficulties are correlated only in the ASD group	0.91 (20/22)
Spek et al. (2017)	ASD (DSM‐IV‐TR; ADI‐R)	ASD: 23 (23) NT: 23 (23)	ASD: 66.1 (6.7) [not reported] NT: 66.3 (6.1) [not reported]	ASD: 107.4 (15.7) NT: 110.3 (13.6)	Processing speed: Processing speed index (WAIS‐III) Working memory: Working memory index (WAIS‐III)	Diagnosis: ASD<NT for processing speed; ASD=NT for working memory^a^	0.77 (17/22)
Torenvliet et al. (2022)	ASD (DSM‐V; ADOS ≥ 8; AQ > 26)	ASD: 88 (54) NT: 88 (54)	ASD: 55.2 (13.9) [31–85] NT: 55.7 (14.4) [30–85]	ASD: 115 (15) NT: 114.4 (14.6)	Episodic memory: Visual memory (WMS‐III) Verbal memory (RAVLT) Working memory: Visual N‐back Processing speed: 2‐choice response task	Diagnosis: ASD<NT for verbal memory; ASD=NT for visual memory, working memory and processing speed Age: 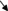 with age for verbal memory visual memory, working memory and processing speed Age × Diagnosis: no interaction observed	0.91 (20/22)
Torenvliet, Groenman, Lever, et al. (2023)	ASD (DSM‐IV; ADOS > 7; AQ > 26)	ASD: 105 (74) NT: 139 (82)	ASD: 47.3 (15.1) [20–79] NT: 45.8 (16.5) [20–77]	ASD: 115.5 (16.8) NT:112.0 (16.4)	Executive functions: *Inhibition*: Go‐NoGo	Diagnosis: ASD=NT for inhibition Age: 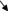 with age for inhibition Age × Diagnosis: no interaction observed	0.95 (21/22)
Torenvliet et al. (2024)	ASD (DSM‐IV/V, ADOS > 7; AQ > 26)	ASD: 202 (135) NT: 247 (144)	ASD: 202 (135) NT: 247 (144)	ASD: 114.9 (16.3) NT:113.3 (16.6)	Episodic memory: Visual memory (WMS‐III) Verbal memory (RAVLT)	Diagnosis: ASD<NT for verbal memory; ASD>NT for visual memory	0.95 (21/22)
Tse et al. (2019)	ASD (DSM‐V)	ASD: 28 (22) NT: 27 (9)	ASD: median = 61 [50–72] NT: median = 63 [50–68]	ASD:113.39 (18.11) NT: 18.89 (11.59)	Processing speed: Processing speed index (WAIS‐IV) Working memory: Working memory index (WAIS‐IV) Visual Working Memory Index (WMS‐V) Episodic memory: Auditory Memory Index (WMS‐V) Visual memory index (WMS‐V) Immediate Memory Index (WMS‐V) Delayed Memory Index (WMS‐V)	Diagnosis: ASD<NT for processing speed and visual working memory; ASD=NT for working memory, auditory memory, visual memory, immediate memory and delayed memory^a^	0.95 (21/22)
Longitudinal (and cross‐sectional) studies							
Torenvliet, Groenman, Radhoe, et al. (2023)	ASD (DSM‐V; ADOS; AQ)	ASD: 128 (84) NT: 112 (72)	ASD: 52.2 (14.2) [24.3–79.3] NT: 55.4 (14.1) [30.4–85.3]	ASD: 114.7 (15.5) NT: 115.4 (16.0)	Episodic memory: Visual memory (WMS‐III) Verbal memory (RAVLT) Working memory: Visual N‐back Executive functions: *Inhibition*: Go‐NoGo *Planning*: Tower of London *Flexibility*: Trail Making Test Processing speed: Choice Response Task	Diagnosis: not reported Change over time: 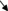 over time for processing speed, inhibition and, 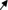 over time for event‐based prospective memory in both groups Change over Time × Diagnosis: no interaction observed Age: 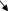 with age for visual memory, verbal memory and processing speed in both groups Age × Diagnosis: no interaction observed	0.82 (18/22)

Abbreviations: ADI: autism diagnostic interview; ADOS: autism diagnostic observation schedule; AQ: autism spectrum quotient; ASD: autism spectrum disorder group; CSS: cross sectional study; DSM‐IV/DSM‐V: Diagnostic and Statistical Manual of Mental Disorders fourth/fifth edition; EQ: empathy quotient; ICD‐10: International Classification of Diseases 10th Revision; IQ: intelligent quotient; LS: longitudinal study; MAA: middle‐aged adults; NT: neurotypical group; SD: standard deviation; SQ: systemizing quotient; YA: young adults.

^a^ Interaction not performed.

### 2.6 Quality Assesment

Two authors (M.B. and S.M.K.) assessed the quality of the included studies using the quantitative scale from QualSyst (Standard Quality Assessment Criteria for Evaluating Primary Research Papers; Kmet et al. 2004). Consensus meetings were used to resolve any disagreements. The quality scores ranged from 0.77 to 0.95, with an average score of 0.88 (SD = 0.06). All studies exceeded the quality threshold of 0.75, indicating high quality. Detailed quality assessments for each study are presented in Tables 2 and 3.

## 3 Results

### 3.1 Study Characteristics

A total of 36 studies were selected. Nine focused on cerebral mechanisms (Braden and Riecken 2019; Dickinson et al. 2022; Hau et al. 2021, 2022; Koolschijn and Geurts 2016; Linke et al. 2020; Raznahan et al. 2010; Tung et al. 2021; van Rooij et al. 2018) (see Table 2). Nineteen studies examined cognitive function (Abbott et al. 2018; Charlton et al. 2023; Davids et al. 2016, 2020; Geurts and Vissers 2012; Geurts et al. 2020; Happé et al. 2016; Harker et al. 2023; Lever et al. 2015; Lever and Geurts 2016a; Powell et al. 2017; Ring et al. 2016, 2020; Spek et al. 2017; Torenvliet et al. 2022, 2024; Torenvliet, Groenman, Lever, et al. 2023; Torenvliet, Groenman, Radhoe, et al. 2023; Tse et al. 2019) (see Table 3). Eight studies addressed both cerebral and cognitive functioning (Bathelt et al. 2020; Baxter et al. 2019; Braden et al. 2017; Kohli et al. 2019; Koolschijn et al. 2017; Pagni et al. 2022; Walsh et al. 2019, 2022) (listed in Table 2).

Fourteen studies employed a cross‐sectional design, exclusively involving older adults (Braden et al. 2017; Charlton et al. 2023; Davids et al. 2016, 2020; Geurts et al. 2020; Geurts and Vissers 2012; Harker et al. 2023; Hau et al. 2021, 2022; Kohli et al. 2019; Linke et al. 2020; Spek et al. 2017; Tse et al. 2019; Tung et al. 2021). Notably, only seven of these studies examined the correlation with age in older adults (Charlton et al. 2023; Davids et al. 2016; Geurts et al. 2020; Geurts and Vissers 2012; Hau et al. 2021, 2022; Kohli et al. 2019), indicating the need for caution. Nineteen cross‐sectional studies looked at how the relationship between age and the outcomes differs between autistic and non‐autistic individuals by including younger adults, either through group comparisons (Baxter et al. 2019; Walsh et al. 2019), a correlational approach (Abbott et al. 2018; Braden and Riecken 2019; Dickinson et al. 2022; Happé et al. 2016; Raznahan et al. 2010; Ring et al. 2016, 2020), or a regression approach^1^ (Bathelt et al. 2020; Koolschijn et al. 2017; Koolschijn and Geurts 2016; Lever et al. 2015; Lever and Geurts 2016a; Pagni et al. 2022; Powell et al. 2017; Torenvliet et al. 2022; Torenvliet, Groenman, Lever, et al. 2023; van Rooij et al. 2018). Although longitudinal studies are warranted for comprehensive understanding and assessment of age‐related differences, as they are the only type of study capable of fully capturing these changes, only three such studies have been published to date (Pagni et al. 2022; Torenvliet, Groenman, Radhoe, et al. 2023; Walsh et al. 2022). Regarding studies on cerebral functioning, six focused on cortical volume (Hau et al. 2022; Pagni et al. 2022; Raznahan et al. 2010; Tung et al. 2021; van Rooij et al. 2018; Walsh et al. 2022), six on cortical thickness (Braden and Riecken 2019; Hau et al. 2022; Kohli et al. 2019; Koolschijn and Geurts 2016; Raznahan et al. 2010; van Rooij et al. 2018), one on gray matter volume (Koolschijn and Geurts 2016), and six on white matter integrity (Braden et al. 2017; Hau et al. 2021, 2022; Koolschijn et al. 2017; Pagni et al. 2022; Walsh et al. 2022). Regarding functional aspects, one study focused on electrophysiological component (Dickinson et al. 2022), and seven focused on the connectivity of brain networks (Bathelt et al. 2020; Baxter et al. 2019; Braden et al. 2017; Hau et al. 2022; Linke et al. 2020; Tung et al. 2021; Walsh et al. 2019). For cognitive functioning, we chose to synthesize the different studies of cognitive aging in autistic adults, focusing on domains that are most vulnerable to the effects of aging: processing speed (e.g., Salthouse 2000; Verhaeghen and Salthouse 1997), executive functions (e.g., Ferguson et al. 2021; Fisk and Sharp 2004), working memory (e.g., Reuter‐Lorenz and Sylvester 2005) and episodic memory (e.g., Luo and Craik 2008). We chose to separate studies involving the updating of information in working memory from those focusing on working memory as a broader construct. Updating is a core component of executive function within working memory (Miyake et al. 2000). However, while all updating tasks involve working memory, not all working memory tasks require active updating. Therefore, we classified tasks that require continuous monitoring and replacement of outdated information under the “updating” section of executive functions, whereas tasks focused on the simple maintenance and manipulation of information were categorized under working memory. Twelve studies examined processing speed (Abbott et al. 2018; Baxter et al. 2019; Davids et al. 2016, 2020; Geurts et al. 2020; Geurts and Vissers 2012; Happé et al. 2016; Powell et al. 2017; Spek et al. 2017; Torenvliet et al. 2022; Torenvliet, Groenman, Radhoe, et al. 2023; Tse et al. 2019), 15 investigated executive functions (Abbott et al. 2018; Bathelt et al. 2020; Braden et al. 2017; Davids et al. 2016; Geurts et al. 2020; Geurts and Vissers 2012; Kohli et al. 2019; Koolschijn et al. 2017; Lever et al. 2015; Powell et al. 2017; Ring et al. 2020; Torenvliet et al. 2022; Torenvliet, Groenman, Lever, et al. 2023; Torenvliet, Groenman, Radhoe, et al. 2023; Walsh et al. 2019), seven explored working memory (Abbott et al. 2018; Davids et al. 2020; Geurts et al. 2020; Geurts and Vissers 2012; Ring et al. 2020; Spek et al. 2017; Tse et al. 2019), and 14 focused on episodic memory (Braden et al. 2017; Charlton et al. 2023; Davids et al. 2020; Geurts and Vissers 2012; Harker et al. 2023; Lever and Geurts 2016a; Pagni et al. 2022; Powell et al. 2017; Ring et al. 2016; Torenvliet et al. 2022, 2024; Torenvliet, Groenman, Radhoe, et al. 2023; Tse et al. 2019; Walsh et al. 2022). Seven studies conducted on cerebral parameters included participants within the range of an intellectual developmental disorder (IDD) (i.e., IQ < 70) (Braden and Riecken 2019; Dickinson et al. 2022; Hau et al. 2021, 2022; Kohli et al. 2019; Linke et al. 2020; Tung et al. 2021). Only five studies provided information on a priori power calculation for determining sample size (Braden et al. 2017; Pagni et al. 2022; Tse et al. 2019; van Rooij et al. 2018; Walsh et al. 2022). Thirty‐one studies included both male and female participants, with an overrepresentation of men, while five studies focused exclusively on men (Baxter et al. 2019; Braden et al. 2017; Raznahan et al. 2010; Spek et al. 2017; Walsh et al. 2019). Thirty‐one studies confirmed the ASD diagnosis using one of the following methods: DSM‐IV (Braden et al. 2017; Braden and Riecken 2019; Dickinson et al. 2022; Geurts et al. 2020; Lever et al. 2015; Lever and Geurts 2016a; Ring et al. 2016, 2020; Spek et al. 2017; Torenvliet, Groenman, Lever, et al. 2023; van Rooij et al. 2018), DSM‐V (Baxter et al. 2019; Davids et al. 2016; Harker et al. 2023; Hau et al. 2021, 2022; Kohli et al. 2019; Linke et al. 2020; Pagni et al. 2022; Torenvliet et al. 2022, 2024; Torenvliet, Groenman, Radhoe, et al. 2023; Tse et al. 2019; Tung et al. 2021; Walsh et al. 2019, 2022), NICE guidelines (Bathelt et al. 2020; Koolschijn et al. 2017; Koolschijn and Geurts 2016) or ICD‐10 (Abbott et al. 2018; Raznahan et al. 2010). Five studies reported a clinical diagnosis of ASD but did not specify the method of assessment (Charlton et al. 2023; Davids et al. 2020; Geurts and Vissers 2012; Happé et al. 2016; Powell et al. 2017). Although the assessment method was not specified, these studies reported a clinical diagnosis of ASD, implying that the diagnoses were made by medical or clinical professionals following established diagnostic criteria, such as DSM‐IV/DSM‐V, ICD‐10, or NICE Guidelines.

The following presentation details studies exclusively examining differences between autistic and non‐autistic older adults, followed by cross‐sectional and longitudinal studies exploring age‐related associations between the two groups.

### 3.2 Impact of Aging in Autism: Insights Into Cerebral Patterns

#### 3.2.1 Structural Aspects

*Brain volume*: Three studies exclusively examined individuals aged over 40 and found either increased (right precentral knob; Hau et al. 2022), similar (total brain volume; Tung et al. 2021), or reduced (hippocampus, Walsh et al. 2022) cortical volume in autistic compared to non‐autistic participants. Cortical volume of the right precentral cortex showed a positive correlation with age in autistic adults, whereas in non‐autistic adults, it showed a negative correlation with age, although these associations did not survive FDR correction (Hau et al. 2022). Three studies included younger to older autistic adults. Two studies found a similar effect of age on subcortical volume in both groups (Pagni et al. 2022; van Rooij et al. 2018). Another highlighted a negative correlation between age and cortical volume in temporal regions, but only in non‐autistic adults (Raznahan et al. 2010). Interestingly, Pagni et al. (2022) employed both cross‐sectional and longitudinal designs and reported findings that diverged between the two approaches. Contrasting with their cross‐sectional findings, the longitudinal study revealed a more pronounced decline in hippocampal volume over 2–3 years in autistic individuals over 40 years compared to non‐autistic adults.

*Cortical thickness*: Three studies focusing on individuals over 40 years of age found no differences in cortical thickness in late adulthood between autistic and non‐autistic individuals (Hau et al. 2022; Kohli et al. 2019; van Rooij et al. 2018). Among the three studies considering both younger to older autistic adults, one reported a stronger negative correlation between age and cortical thickness in frontal, temporal, and parietal regions in autistic individuals compared to non‐autistic adults (Braden and Riecken 2019). Another found a similar effect of age in both groups (Koolschijn and Geurts 2016), while the third observed a negative correlation with age in frontal and temporal regions only in non‐autistic individuals (Raznahan et al. 2010).

*Gray matter volume*: A single study (Koolschijn and Geurts 2016) compared the whole brain gray matter volume and found a similar effect of age on gray matter volume in both autistic and non‐autistic adults.

*White matter integrity*: Among studies focusing exclusively on older adults, two found no differences in white matter characteristics between autistic and non‐autistic participants (Braden et al. 2017; Hau et al. 2021), while two studies reported reduced white matter integrity in autistic adults only (Hau et al. 2022; Walsh et al. 2022), though one study did not survive FDR correction (Hau et al. 2022). When including individuals from younger to older ages, Koolschijn et al. (2017) showed an effect of age on white matter integrity in autistic adults relative to non‐autistic adults, and noted that greater white matter microstructural changes were associated with increased cognitive variability in autistic individuals. Conversely, Pagni et al. (2022) found that although autistic adults had altered white matter integrity compared to non‐autistic adults, they reported a similar effect of age in both groups. Using a longitudinal design, Pagni et al. (2022) also observed age‐related decline in white matter integrity over 2–3 years, regardless of diagnostic group. In the same cohort as the previous study, Walsh et al. (2022) found that baseline alterations in hippocampal white matter integrity were negatively correlated with delayed visual memory change over 2–3 years in autistic individuals.

#### 3.2.2 Functional Aspects

*Electrophysiological component*: One study using EEG with both young and older adults reported a stronger negative correlation between age and Peak Alpha Frequency during resting state in autistic participants, associated with lower levels of cognitive ability, compared to non‐autistic participants (Dickinson et al. 2022).

*Brain network connectivity*: Considering only autistic adults first, two fMRI studies found hyperactivity (Bathelt et al. 2020) or less deactivation (Baxter et al. 2019) in the default mode network (DMN) in older autistic adults compared to non‐autistic older adults. However, Braden et al. (2017) reported no differences between autistic and non‐autistic older adults in the DMN, working memory network, or salient network functional connectivity. Additionally, they observed reduced functional connectivity of the cortico‐striato‐thalamo‐cortical neural network in older autistic adults compared to non‐autistic adults during task switching between low and high working memory loads. Furthermore, reduced connectivity was observed in the anxiety network, encompassing a network of regions associated with anxiety responses across diverse anxiety disorders in non‐autistic individuals (Tung et al. 2021) and in the sensorimotor system (Hau et al. 2022; Linke et al. 2020) in autistic older adults. While Linke et al. (2020) found no correlation between age and sensorimotor connectivity in both groups, Hau et al. (2022) reported a positive correlation between age and sensorimotor connectivity in autistic older adults and a negative correlation in non‐autistic older adults, although not surviving FDR correction. Three studies included younger to older adults. Bathelt et al. (2020) observed a similar effect of age on DMN connectivity and fronto‐parietal network connectivity in both groups. However, they found an effect of age on visual network connectivity only in non‐autistic individuals. Baxter et al. (2019) observed that despite similar cognitive task performance, young and older participants in both groups used distinct neural network activations. Lastly, Walsh et al. (2019) found a pattern of hypoconnectivity in the dorsolateral prefrontal cortex within the executive network in autistic adults compared to non‐autistic adults, with greater hypoconnectivity observed in older autistic individuals.

### 3.3 Aging in Autism: Profiles of Cognitive Changes

#### 3.3.1 Processing Speed

Six studies focused exclusively on older adults and found either reduced (Spek et al. 2017; Tse et al. 2019) or preserved (Davids et al. 2016, 2020; Geurts et al. 2020; Geurts and Vissers 2012) processing speed abilities in autistic compared to non‐autistic older adults, with no correlation with age (Davids et al. 2016) or effect of age in either group (Geurts et al. 2020; Geurts and Vissers 2012). A study by Baxter et al. (2019) examined both autistic and non‐autistic participants across two age groups (young vs. older) and found that, although age did not have a significant effect, autistic adults exhibited lower processing speed abilities compared to non‐autistic adults, irrespective of age group. Two other studies (Powell et al. 2017; Torenvliet et al. 2022) found a similar effect of age on processing speed abilities in both autistic and non‐autistic adults. Conversely, two studies reported a differential relationship with age, with positive correlations between age and processing speed measures in autistic participants only (Abbott et al. 2018; Happé et al. 2016). Furthermore, Torenvliet, Groenman, Radhoe, et al. (2023) employed a longitudinal design and observed a similar decline of processing speed over 6–10 years between autistic and non‐autistic adults.

#### 3.3.2 Executive Functions (EF)

For clarity's sake, the different studies will be divided according to the main EF components sensitive to age effects (e.g., Ferguson et al. 2021; Fisk and Sharp 2004).

*Self‐reported executive capacity*: Two studies (Davids et al. 2016; Geurts et al. 2020) found that autistic adults over the age of 50 reported more executive difficulties on the Behavior Rating Inventory of Executive Function scale (Gioia et al. 2002) compared to non‐autistic adults.

*Updating*: Four studies investigated updating abilities, all of which used the N‐back task. Among studies focusing only on older individuals, Braden et al. (2017) found no difference between autistic and non‐autistic participants. Two studies included both younger to older adults. One of them (Torenvliet et al. 2022) observed a similar effect of age on updating abilities between autistic and non‐autistic adults. In contrast, another study (Lever et al. 2015) found a linear effect of age in non‐autistic individuals, whereas both linear and quadratic effects of age were observed in autistic individuals, interpreted by the authors as age‐related increases being associated with better performance in autistic adults. Additionally, one longitudinal study (Torenvliet, Groenman, Radhoe, et al. 2023) found no age‐related decline in updating abilities over 6 to 10 years in either group.

*Inhibition*: Six studies examined inhibition abilities. One study, focusing only on older adults, reported lower scores in autistic individuals compared to non‐autistic adults (Kohli et al. 2019, D‐KEFS Color Word Interference task). Four studies, including younger to older adults, were conducted. One study did not report group differences or the effect of age across groups (Bathelt et al. 2020, Flanker task), while another found no differences between autistic and non‐autistic adults but did not provide information on the possible effect of age (Koolschijn et al. 2017, Flanker task). Additionally, the other two cross‐sectional studies found a similar correlation with age (Abbott et al. 2018, Hayling test) or the effect of age on inhibition abilities between autistic and non‐autistic adults (Torenvliet, Groenman, Lever, et al. 2023, Go‐NoGo task). A longitudinal study by Torenvliet, Groenman, Radhoe, et al. (2023) observed an age‐related decline in inhibition abilities over 6–10 years, with no differences in this evolution between autistic and non‐autistic adults (Go‐NoGo task).

*Flexibility*: Eight studies were conducted on flexibility abilities. Four studies focusing exclusively on older adults found either no significant differences in flexibility abilities between autistic and non‐autistic groups (Geurts et al. 2020, Wisconsin Card Sorting Test; Geurts and Vissers 2012, Modified Card Sorting Test and Trail Making Test) or reported greater difficulties in the autistic group (Braden et al. 2017, Wisconsin Card Sorting Test; Kohli et al. 2019, D‐KEFS Trails task), with no correlation with age observed in either group. Three additional studies examined differential age effects, but one of them (Ring et al. 2020, Color Trails test) did not report the group differences. One study found a positive correlation between age and flexibility abilities in autistic adults only (Abbott et al. 2018, Trail Making Test), while another reported an effect of age only in autistic adults (Powell et al. 2017, Trail Making Test). Torenvliet, Groenman, Radhoe, et al. (2023) conducted a longitudinal study that found no age‐related decline in flexibility abilities (Trail Making Test) over 6–10 years in either group.

*Planning*: Six studies investigated planning abilities. Three studies were conducted on older adults aged over 50 (Davids et al. 2016, Tower of London and Zoo Map tests; Geurts et al. 2020, Tower of London and Zoo Map tests; Geurts and Vissers 2012, Tower of London) and found no significant differences in planning abilities between autistic and non‐autistic older individuals, and no effect of age in either group. Two studies included younger to older adults. Walsh et al. (2019) found no differences in planning abilities between age groups (young vs. older adults) in either autistic or non‐autistic individuals (Tower of London task). Abbott et al. (2018) reported no difference in the correlation between age and performance between autistic and non‐autistic adults (Zoo Map and Key Search tests). One longitudinal study (Torenvliet, Groenman, Radhoe, et al. 2023, Tower of London) found no age‐related decline over 6–10 years in either group.

#### 3.3.3 Working Memory (WM)

Five studies were conducted solely on adults over 50 and found lower visuo‐spatial WM performance in autistic compared to non‐autistic adults (Geurts and Vissers 2012; Tse et al. 2019), while no differences were observed between groups for verbal WM (Davids et al. 2020; Geurts et al. 2020; Spek et al. 2017; Tse et al. 2019), and no effect of age on visuo‐spatial or verbal WM abilities was found in either group (Geurts et al. 2020; Geurts and Vissers 2012). When including younger to older adults, two studies reported mixed results: one found no difference between autistic and non‐autistic adults in the correlation between age and WM performance (audio‐verbal, Abbott et al. 2018), while another observed a negative correlation between age and working memory performance only in non‐autistic individuals (both audio‐verbal and visuo‐spatial, Ring et al. 2020). Additionally, the latter study demonstrated through regression analysis that autistic adults relied more on flexibility abilities compared to their non‐autistic counterparts.

#### 3.3.4 Episodic Memory

Among studies focusing only on older adults, four found no significant differences between autistic and non‐autistic individuals in either immediate or delayed verbal memory (Braden et al. 2017; Geurts and Vissers 2012; Harker et al. 2023; Tse et al. 2019). One of these studies reported a similar effect of age on immediate verbal memory in both older groups (Geurts and Vissers 2012). For visual memory, results were mixed: no differences were found in immediate and delayed visual memory between autistic and non‐autistic older adults (Davids et al. 2020; Geurts and Vissers 2012; Tse et al. 2019). However, both older groups exhibited a similar effect of age on delayed visual memory, whereas the effect of age on immediate visual memory appeared stronger in autistic individuals (Geurts and Vissers 2012). Five studies included younger to older adults. One found that autistic adults performed worse than non‐autistic adults on verbal memory tasks but better on visual memory tasks, but the effect of age was not reported (Torenvliet et al. 2024). Four studies identified a comparable effect of age on memory performance in both groups, including immediate (Lever and Geurts 2016a; Powell et al. 2017; Torenvliet et al. 2022) and delayed verbal memory (Lever and Geurts 2016a; Pagni et al. 2022; Torenvliet et al. 2022), as well as immediate (Torenvliet et al. 2022) and delayed visual memory (Lever and Geurts 2016a; Torenvliet et al. 2022). However, two studies found an effect of age on immediate verbal memory (Pagni et al. 2022) and on immediate visual memory (Lever and Geurts 2016a), exclusively in non‐autistic individuals. Additionally, three studies employed a longitudinal design. Pagni et al. (2022) observed a greater decline over 2–3 years in immediate verbal memory among autistic adults but found no significant differences in the evolution between groups for delayed verbal memory. Similarly, Walsh et al. (2022) reported a greater decline over 2–3 years in delayed visual memory among autistic adults, yet noted no significant differences in changes over time between groups for immediate visual memory. In contrast, Torenvliet, Groenman, Radhoe, et al. (2023) found no significant differences in age‐related change over 6–10 years between groups for either immediate or delayed verbal and visual memory.

Two studies explored additional aspects of episodic memory. One study found a negative correlation between age and memory abilities for relational information in non‐autistic adults but not in autistic adults (Ring et al. 2016). Another study, using subjective measures with a questionnaire, reported more pronounced episodic memory complaints in autistic older adults compared to non‐autistic older adults (Charlton et al. 2023). Additionally, older age was correlated with fewer memory complaints in the autistic group, whereas no such correlation was found in the non‐autistic group (Charlton et al. 2023).

## 4 Discussion

This review is intended to summarize, for the first time, research on non‐social cognitive functions and both structural and functional brain changes in older autistic adults, to better understand the impact of aging on the growing population of middle‐aged and older autistic adults. We highlighted the plurality of results in the literature, which, as it stands, does not allow forming a clear cerebral and cognitive aging pattern in autism.

Before summarizing the studies, we would like to highlight methodological limitations in the research designs that constrain the interpretations and conclusions that can be drawn. As in neurocognitive aging research more broadly, most studies examining aging in autism rely on cross‐sectional designs that compare different age groups at a single time point. Although these studies provide insights into differences between autistic and non‐autistic individuals and explore correlations between age and specific outcomes, they do not directly capture aging trajectories. Their findings, often interpreted as evidence of age‐related decline or improvement in line with the three autism aging hypotheses: parallel, accelerated, or slowed aging, should therefore be interpreted cautiously. Longitudinal studies are more appropriate for understanding aging processes in autism. This limitation of cross‐sectional studies is particularly significant in aging research on autism, as differences between cohorts within the autistic population can influence findings. Cohort effects must be accounted for when comparing younger and older autistic adults in cross‐sectional studies, as access to intervention, changes in autism recognition, and diagnostic criteria over time may affect comparability between these age groups. This is supported by varying results reported in longitudinal versus cross‐sectional studies (Pagni et al. 2022). Therefore, differences between younger and older autistic adults may not solely reflect age but could also be shaped by these varying factors. Another critical concern is the potential selection bias. Most studies focus on high‐functioning individuals, many of whom were diagnosed in adulthood, which does not fully represent the broader autistic population. The selection bias is particularly significant in cross‐sectional studies. Individuals experiencing accelerated aging may be underrepresented due to recruitment challenges, exclusion criteria, or impairments that hinder participation. Additionally, some individuals in this subgroup may no longer be living, further skewing the findings toward those with more typical aging trajectories. This underrepresentation highlights the need for careful interpretation of the existing literature. Recognizing this limitation, we present the main findings for each outcome in the discussion section as interpreted in the original studies, while encouraging readers to critically assess the constraints of cross‐sectional designs.

Regarding cerebral parameters, particularly structural aspects, only 11 studies have investigated morphological indicators in aging autism. These studies focused on a limited number of cerebral domains. Although several studies have examined white matter integrity, none have explored how white matter volume evolves with aging in autism, highlighting the need for further investigation. Findings from cross‐sectional studies have been inconsistent, often interpreted as supporting the accelerated, parallel, or safeguard hypotheses. In contrast, the only longitudinal study on structural cerebral parameters showed an accelerated aging pattern for cortical brain volume and a parallel aging trajectory for white matter integrity. The similarities between alterations observed in non‐autistic aging and those present in autism, combined with the lack of studies, and particularly longitudinal research, on aging in autism, underline the need for further research to better understand structural changes in this population. On functional aspects, neuroimaging studies with older autistic adults are limited, with only eight studies conducted. Cross‐sectional studies have produced varied results, which have been interpreted in terms of accelerated, parallel, or slowed aging compared to non‐autistic adults. However, in the absence of longitudinal studies on functional parameters, it remains challenging to accurately assess age‐related changes between groups, highlighting the need for further investigation. Furthermore, most studies focused on neural network connectivity, with none examining brain activity, underscoring the need for research to examine the activity of regions specifically sensitive to both autism and aging, particularly frontal regions, and their implications in cognitive tasks. Additionally, it is crucial to study other electrophysiological indicators known to be sensitive to aging (e.g., MMN, Cheng et al. 2013; P300, van Dinteren et al. 2014; FN400, Wolk et al. 2009) in more detail. Given the cerebral reorganization mechanisms highlighted in non‐autistic aging (e.g., Hemispheric Asymmetry Reduction in Older Adults, Cabeza 2002; Posterior–Anterior Shift in Aging, Dennis and Cabeza 2008) alongside the cerebral specificities observed in autistic adults, exploring the evolution of these mechanisms over time would be particularly interesting.

Research on cognitive aging in autism presents varied findings. For processing speed, cross‐sectional studies interpret their findings in alignment with either the parallel or safeguard hypotheses, while longitudinal research highlights a similar aging process between autistic and non‐autistic individuals. In executive functions, cross‐sectional studies interpreted their results as showing parallel aging for inhibition and planning capacities. However, findings related to updating and flexibility abilities are inconsistent, with interpretations suggesting an aging process that could be accelerated, parallel, or slower compared to non‐autistic individuals. In contrast, the only longitudinal study conducted revealed a similar aging trajectory between groups for updating, inhibition, flexibility, and planning abilities. Working memory results of cross‐sectional studies vary depending on study type and stimulus modality. For verbal working memory, the authors interpret the findings as indicating similar working memory abilities with age in both autistic and non‐autistic adults. In contrast, visuo‐spatial working memory findings are interpreted as showing lower capacity in older autistic adults, although the decline in visuo‐spatial working memory abilities with age is considered less pronounced in this group. However, no longitudinal studies have been conducted to examine actual changes in working memory capacity over time. For episodic memory, research indicates divergent aging profiles according to the modality (verbal vs. visual). Cross‐sectional studies on verbal memory are interpreted as indicating aging trajectories ranging from parallel to slowed aging. These variations may reflect differences in the assessment methods, such as immediate versus delayed recall. The parallel aging hypothesis is often supported by studies using delayed verbal episodic memory assessments, while immediate verbal episodic memory assessments show mixed patterns of either parallel or slowed aging. For visual memory, findings reveal aging profiles ranging from accelerated to slowed aging, which may also be influenced by the assessment method. Immediate visual memory assessments are interpreted as supporting all three aging hypotheses, even when similar tasks are employed, whereas delayed visual memory assessments are typically associated with parallel aging. Only three studies have used a longitudinal design, and their findings are contradictory. For verbal memory, results suggest either a parallel or accelerated aging trajectory for immediate verbal memory assessments and a parallel aging trajectory for delayed verbal memory assessments. For visual memory, immediate visual memory assessments indicate a parallel aging process, while delayed visual memory assessments show either accelerated or parallel aging trajectories.

Although existing research is limited, cross‐sectional studies generally report that aging in autism tends to occur at a similar or slower rate than in non‐autistic adults, while longitudinal studies more often report parallel or accelerated patterns of cerebral and cognitive aging in specific cerebral parameters and cognitive functions. However, caution is warranted when interpreting these conclusions, as the existing literature is limited, and further research is needed to confirm this. According to the safeguard hypothesis, autistic adults may develop strategies to address challenges in older age due to early life difficulties. Alternatively, the relationship between age and cognitive outcomes might be less pronounced in autistic individuals, as their processing speed slows from childhood and they tend to prioritize precision over speed (Zapparrata et al. 2022). Additionally, there may be differences between how autistic individuals function in laboratory settings versus real‐world environments, which makes it challenging to fully support and validate this conclusion outside of controlled settings. Compensatory strategies that older autistic individuals may employ in laboratory settings could be effective in controlled environments, but might not translate as well to real‐world situations, or vice versa. However, the safeguard hypothesis is challenged by findings from Vivanti et al. (2025), who reported that older autistic adults (≥ 65 years) are 18.15 times more likely to receive a dementia diagnosis than younger autistic adults. This suggests that prior support for the safeguard hypothesis may instead reflect biased sampling rather than a genuine protective effect. Additionally, evidence supporting the safeguard hypothesis has only been reported in cross‐sectional studies, which are limited in capturing the trajectory of cerebral and cognitive aging. In contrast, longitudinal studies generally suggest similar or more pronounced aging patterns in autistic individuals.

Besides study designs (cross‐sectional or longitudinal), the variability in study results may be due to differences in study protocols, small sample sizes, and the specific tests used. Given the limited literature on cognitive and cerebral aging in autism, it is important to recognize that variations in findings may stem from methodological differences, such as the specific measurement techniques or methodologies used to assess the same cognitive domain across studies. Some of the observed results may be artifacts of these measurement procedures, which could prevent definitive conclusions from being drawn at this stage. Additionally, internal factors (e.g., participant age, autism severity, IQ, co‐occurring conditions) and external influences (e.g., lifestyle, educational level, socio‐demographic, sensory challenges, psycho‐affective variables) could contribute to this diversity. The inclusion of autistic adults with IDD in several studies also adds to the variability (Braden and Riecken 2019; Dickinson et al. 2022; Hau et al. 2021, 2022; Kohli et al. 2019; Linke et al. 2020; Tung et al. 2021). Furthermore, the literature has identified a number of factors that may help maintain cognitive abilities during aging, known as cognitive reserve factors (Stern 2002). These elements, such as education, physical activity, social engagement, and leisure activities, may influence cognitive aging differently for each individual, potentially accounting for the variability in study results. Moreover, autism characteristics and co‐occurring conditions are often linked with psychotropic medication use (Chamak and Bonniau 2016; Nylander et al. 2018), which, despite its positive impact on behavior (Vasa et al. 2014; Zhou et al. 2021), lacks sufficient research on its long‐term effects on cerebral and cognitive functioning (Esbensen, Greenberg, et al. 2009; Jobski et al. 2017). These variables should be considered to better understand the cerebral and cognitive aging process in autism.

This review supports the previous systematic review (Tse et al. 2022) and meta‐analysis (Wang et al. 2024), which reveal a complex picture of cognitive and cerebral aging in autism. Tse et al.'s (2022) initial review on cognitive functions missed some later research, which our review incorporates. Wang et al. (2024) found significant challenges for autistic individuals in flexibility, working memory (particularly visual memory), and subjective executive functions, with similar performance to non‐autistic individuals in planning and episodic memory. Their meta‐analysis, while insightful, does not address age effects, preventing conclusions about accelerated, parallel, or slowed aging. Our review confirms and extends the structural differences they observed by incorporating functional imaging studies, which are crucial for understanding cognitive impacts. Functional imaging can reveal age‐related changes in brain activity, identifies neural mechanisms of cognitive decline, and detects patterns missed by structural imaging, thereby enhancing our understanding of cerebral aging in autism. However, the lack of longitudinal research in this area significantly limits our ability to fully understand these processes.

Several limitations in the reported studies should be noted. Most research has predominantly involved autistic adults with average IQs and less severe characteristics, with only a few exceptions (Braden and Riecken 2019; Dickinson et al. 2022; Hau et al. 2021, 2022; Kohli et al. 2019; Linke et al. 2020; Tung et al. 2021). Further research is needed to explore aging within this subgroup of autistic individuals, including those with lower cognitive abilities, to better understand aging effects across these subpopulations. However, achieving this goal presents significant logistical and methodological challenges, requiring adjustments in experimental designs and neuropsychological assessments to effectively evaluate memory and executive functions in individuals with varying levels of cognitive skills. Moreover, only twenty‐five studies involved autistic adults over 65, restricting our understanding of aging in autism. While we acknowledge the complexity of including older autistic adults in studies, we believe that as the global aging population continues to grow, the number of aging autistic adults will also increase, creating more opportunities for future studies to include a larger sample of this population, helping to better address this gap in research. Though they provided valuable insight into the field, several studies in the review were conducted by the same research groups and may involve overlapping participants, potentially introducing bias toward specific autism aging hypotheses (group 1: Bathelt et al. 2020; Koolschijn et al. 2017; Koolschijn and Geurts 2016; Lever et al. 2015; Lever and Geurts 2016a; group 2: Torenvliet et al. 2022, 2024; Torenvliet, Groenman, Lever, et al. 2023; Torenvliet, Groenman, Radhoe, et al. 2023; group 3: Baxter et al. 2019; Braden et al. 2017; Braden and Riecken 2019; Harker et al. 2023; Pagni et al. 2022; van Rooij et al. 2018; Walsh et al. 2019, 2022; group 4: Hau et al. 2021, 2022; Kohli et al. 2019; Linke et al. 2020; Tung et al. 2021; group 5: Ring et al. 2016, 2020). Additionally, existing longitudinal studies are limited in number and typically conducted over short durations. Although challenging, it would be valuable to pursue further research with longer follow‐up periods to minimize cohort effects observed in cross‐sectional studies and provide more reliable comparisons.

In conclusion, the study of cerebral and cognitive aging in autism revealed varied aging patterns. Despite increased research, there remains a significant gap, especially regarding cerebral aging. Addressing aging in autism is crucial for tailoring support to older autistic adults and improving diagnostic accuracy for middle‐aged and older individuals. A deeper understanding of these aging processes will not only aid in identifying neurodegenerative diseases more effectively but also enhance support for older autistic adults by facilitating the adaptation of interventions and the development of more precise cognitive remediation programs tailored to their specific needs and abilities. Continued research is essential to evaluate how aging affects autistic symptomatology and to provide appropriate support to an ever‐growing proportion of the population.

## Author Contributions

**Marine Bessé:** writing – original draft, conceptualization, methodology. **Shasha Morel‐Kohlmeyer:** writing – review and editing, supervision, conceptualization, validation. **Emmanuelle Houy‐Durand:** conceptualization. **Philippe Prévost:** writing – review and editing, conceptualization. **Laurie Tuller:** writing – review and editing, conceptualization. **Badiâa Bouazzaoui:** writing – review and editing, conceptualization. **Laurence Taconnat:** writing – review and editing, conceptualization. **Julia Capdeville:** writing – review and editing, methodology. **Lucie Angel:** writing – review and editing, supervision, conceptualization, validation. **Marie Gomot:** writing – review and editing, supervision, conceptualization, validation.

## Conflicts of Interest

The authors declare no conflicts of interest.

## Data Availability

Data sharing not applicable to this article as no datasets were generated or analysed during the current study.

## Endnotes

^1^
As employed by the authors, regression analyses are systematically referred to by the expression “age effect” in this review.
